# A new cell-laden 3D Alginate-Matrigel hydrogel resembles human breast cancer cell malignant morphology, spread and invasion capability observed “*in vivo*”

**DOI:** 10.1038/s41598-018-23250-4

**Published:** 2018-03-28

**Authors:** Marta Cavo, Marco Caria, Ilaria Pulsoni, Francesco Beltrame, Marco Fato, Silvia Scaglione

**Affiliations:** 1 National Research Council (CNR) – IEIIT Institute, Genoa, 16149 Italy; 2 Department of Biophysical and Electronic Engineering (DIBRIS), University of Genoa, Genoa, 16145 Italy; 3 React4life S.r.l, Genoa, 16100 Italy

## Abstract

Purpose of this study was the development of a 3D material to be used as substrate for breast cancer cell culture. We developed composite gels constituted by different concentrations of Alginate (A) and Matrigel (M) to obtain a structurally stable-in-time and biologically active substrate. Human aggressive breast cancer cells (i.e. MDA-MB-231) were cultured within the gels. Known the link between cell morphology and malignancy, cells were morphologically characterized and their invasiveness correlated through an innovative bioreactor-based invasion assay. A particular type of gel (i.e. 50% Alginate, 50% Matrigel) emerged thanks to a series of significant results: 1. cells exhibited peculiar cytoskeleton shapes and nuclear fragmentation characteristic of their malignancy; 2. cells expressed the formation of the so-called invadopodia, actin-based protrusion of the plasma membrane through which cells anchor to the extracellular matrix; 3. cells were able to migrate through the gels and attach to an engineered membrane mimicking the vascular walls hosted within bioreactor, providing a completely new 3D *in vitro* model of the very precursor steps of metastasis.

## Introduction

Breast cancer is the most common cancer in women across most ethnic groups and one of the leading causes of cancer-related deaths worldwide^1–3^. Mortality is mainly associated with the development of metastases - the spread of a tumour from its primary site to other parts of the body - than to symptoms strictly related to the main lesion^4,5^. Thus, a deeper understanding of the pathways that give rise to metastasis is one of the key challenges for developing new therapies to fight breast cancer^6–8^.

Metastasis is a complex and multistep process: in order to generate secondary tumours, cells must detach from their primary site, enter within the systemic circulation, establish contacts with the endothelium^9^, adhere to the vascular walls^10^ and finally transmigrate across the endothelial layers^11^ as single cells or clusters^12,13^.

Different sub-processes acting at the cellular level guide each of these steps: several key stages of metastasis - including invasion, intravasation, and extravasation - are thought to involve Extra-Cellular Matrix (ECM) degradation and remodelling^14^. Cancer cells contribute to matrix degradation through actin-rich subcellular protrusions known as invadopodia^15^. Invadopodia consists of an actin-rich core surrounded by a number of important protein components, including cytoskeletal modulators, adhesion proteins, scaffolding proteins, and signaling molecules^16^.

Traditionally, cancer biology research has involved *in vitro* analysis of cell behaviour predominately using two-dimensional (2D) cell cultures and *in vivo* animal models^17,18^: in detail, 2D models are routinely used as initial systems for evaluating the effectiveness of molecules as potential therapeutic drugs; this initial screening precedes animal studies before advancing to human clinical trials^19^. It is well known that these two categories of models differ widely, especially in the microenvironment surrounding cells^20–22^. Differences between these models and human malignancies are also known: the dissimilarities in cell behaviour between 2D cultures and real tumours derive from changes in gene expression originated from the different interactions to which cells are subjected within a 2D microenvironment if compared to a more natural 3D^23,24^. A striking example of that is represented by the unequal nutrient concentration to which cells are exposed: in 2D cultures cells are uniformly exposed to nutrients, while *in vivo* the concentration of soluble factors influencing cell proliferation is characterized by spatial gradients that play a vital role in biological differentiation, organ development, determination of cell fate and signal transduction^25,26^.

Several phenomena, such as metastasis process and tissue organization, cell motility and proliferation, have been proven to be regulated by mechanical interactions with the surrounding microenvironment^27–29^. On the other side, animal models of metastasis include human–mouse xenografts and genetically engineered mice, resulting in a lack of a single and worldwide recognized metastasis model^30^. All these gaps may lead to inaccurate assessment of cancer biology, presenting a clear need for more standardized and realistic models for the study of disease mechanisms, drug efficacy and cell characterization studies^31,32^.

Trying to fill these gaps, a wide range of new 3D *in vitro* models is recently emerging to better mimic the physiological human context. These systems, including cell spheroids and solid three-dimensional (3D) cell cultures in an artificial ECM, have numerous potential advantages over existing models, e.g. increased reproducibility, precise control over cultivation conditions and incorporation of human cells^21,33,34^. Moreover, they should conduce to more systematic and quantitative investigations than *in vivo* models.

In that context, hydrogels have gained attention thanks to their high biocompatibility and efficient oxygen and nutrient transportation; however, many current hydrogel-based tumour models still lack crucial features such as a biologically relevant composition and/or an appropriate volume to best mimic a human tumour *in vivo*^17,20,35^.

Proposing to take steps in the advancement of a 3D structure in which breast cancer cells can grow and manifest their aggressive and metastatic potential, we have analysed benefits and drawbacks of several already proposed materials. Among them, we focused our attention on two basic materials, i.e. Alginate and Matrigel, having different but complementary characteristics, with the final aim to give rise to a new category of composites able to be both structurally stable over time and biologically permissive.

Alginate is a good candidate for the accomplishment of a 3D structure stable over a prolonged time^36^, necessary for the realization of an *in vitro* model for pharmacological tests. Alginate can be easily arranged in a 3D gel-like structure and the mechanical properties of the resultant gel can be precisely tuned via calcium ions-mediated crosslinking^37,38^. In a previous study, we compared viability, proliferation rates and organization form of lowly aggressive breast cancer cells (i.e. MCF-7 cell line) when embedded in 3D alginate gels with different stiffness, finally defining the most suitable amounts of alginate and calcium to enhance cell activity^29^. This alginate-based model resulted appropriate for the culture of lowly aggressive cells, that both in 2D and in 3D maintain a pretty round morphology and a cluster-like organization^39^, while a much more permissive environment becomes necessary when invasive phenomena need to be studied.

Matrigel is a soluble and sterile extract of basement membrane proteins derived from the EHS tumour that forms a 3D gel at 37 °C^40^, known to enhance cell biological events and to allow cells expressing some key features reflecting their inner malignancy, such as a more elongated shape related to their invasion capability^39^; however, its structural weakness allows using it only in monolayer or thin gel conformations, mainly for invasion assays^41^. Moreover, because of its permissiveness, Matrigel is adopted for short-term analysis, ranging from few hours^42^ to 4 days^43^.

From these considerations, the idea of new composites able to join the advantages of the two bulk materials was born, with the final aim to obtain a structurally performing and biologically permissive material to be adopted for the 3D culture of aggressive breast cancer cells.

In this work, a new sperimental protocol to obtain 3D cell-laden composite materials with a volume comparable to real tumours was developed; alginate and Matrigel were mixed at different percentages and the resultant structural stability was verified. Once the most appropriate concentrations were found, highly metastatic breast cancer cells (i.e. MDA-MB-231) were embedded within the gels.

Cell viability and proliferation were firstly checked and monitored to evaluate the cytocompatibility of the new materials. Then, we focused our attention on cell morphological features: both nuclei mutations and cytoskeletal features, expressive of cell malignancy, were monitored up to 7 culture days. These morphological changes were quantitative extrapolated to evaluate the statistically significant differences.

To cross-correlate cell morphology and invasion capability - as expression of their malignancy - tumour gels were finally placed in an innovative multi-organ bioreactor-based set-up by React4life S.r.l.

Cell ability to migrate through the gels and escape from them was observed as preliminary step of a simplified but of great potential *in vitro* metastasis model.

## Results

### Composite hydrogel assessment

**Figure 1:**
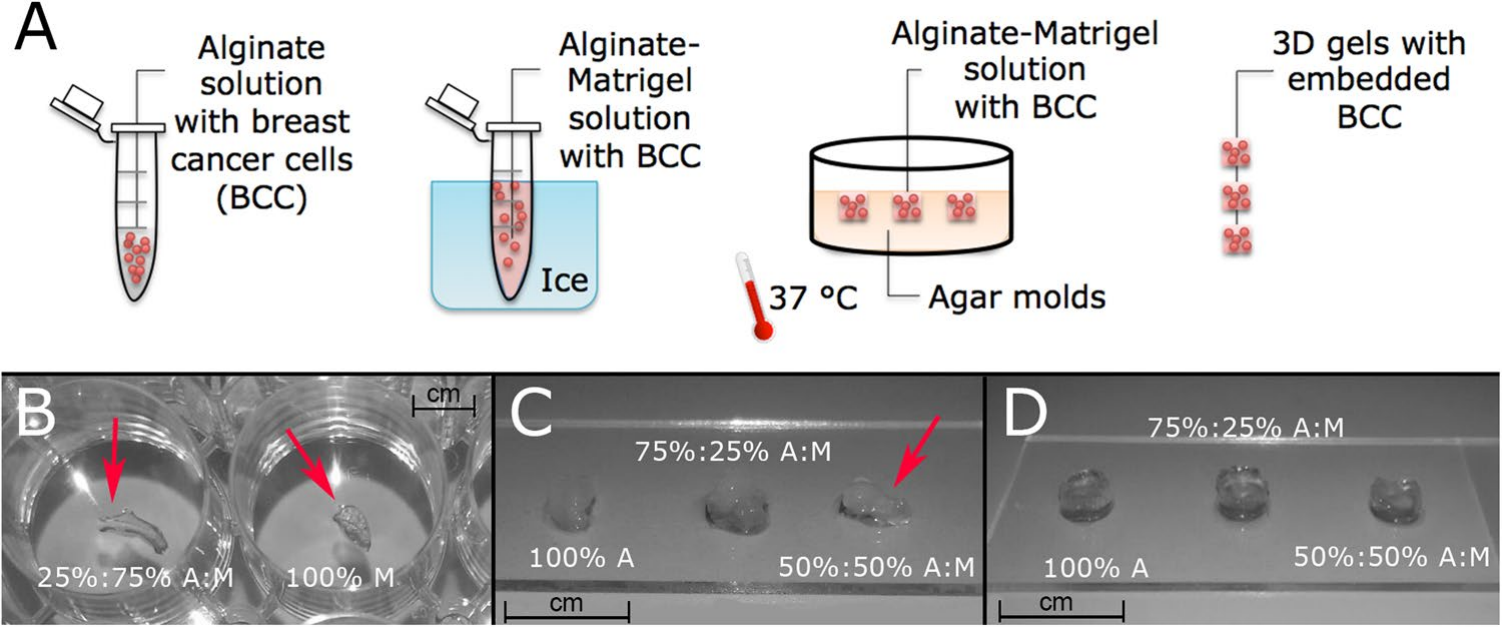
Composite cell-laden gel development. (**A**) Schematic description of the protocol used to produce 3D cell-laden Alginate-Matrigelcomposite gels: breast cancer cells MDA-MB-231 are seeded first in liquid alginate and then Matrigel is added working on ice. The cell-laden solution is transferred into Agar molds enriched with CaCl2 ions at 37 °C for to allow gelation. Then, 3D gels are removed by molds and trasferred into plates with culture media. (**B**) Gels composed by 25%:75% A:M and 100% M ratios resulted too soft and not handy. (**C**) a concentration of 2 million cells/ml caused a fast degradation of 50%:50% A:M gels, thus it was reduced to 1 million cells/ml. (**D**) finally, structurally compact gels were obtained.

Hydrogel initial compositions (i.e. 100% A, 75%:25% A:M, 50%:50% A:M, 25%:75% A:M, 100% M) were firstly assessed from a structural point of view. Hydrogels belonging to 25%:75% A:M and 100% M categories did not show a proper robustness and structural stability and thus were immediately excluded (Fig. 1, panel B). The other three categories were chosen as substrates for 3D cell culture. Initially, gels of 100 μl were loaded with 200.000 cells each one; however, this concentration caused a fast degradation of the 50%:50% A:M category (Fig. 1, panel C). To our knowledge of the current literature, this behaviour may be caused by the action of the metal-protease MT-MMP, necessary for cell proliferation and for the integrin-mediated invasion process. To solve these issues, already expressed in some works in literature^44^, we adopted two different strategies: first, cell density was reduced from 200.000 to 100.000 per gel, ensuring a still good cell-to-cell contact. Secondly, we functionalized the bottom of the culture plate with a thin layer of Matrigel before moving gels into, as suggested by Lee *et al*.^45^.

Under these conditions, stable gels for the whole experimental period were obtained (Fig. 1, panel D).

### Hydrogel mechanical characterization by AFM

Using AFM nanoindentation technique, the Young modulus of each hydrogel was measured at the sub-micrometer scale, the same length scale of the actual cell sensing^46^.

**Figure 2:**
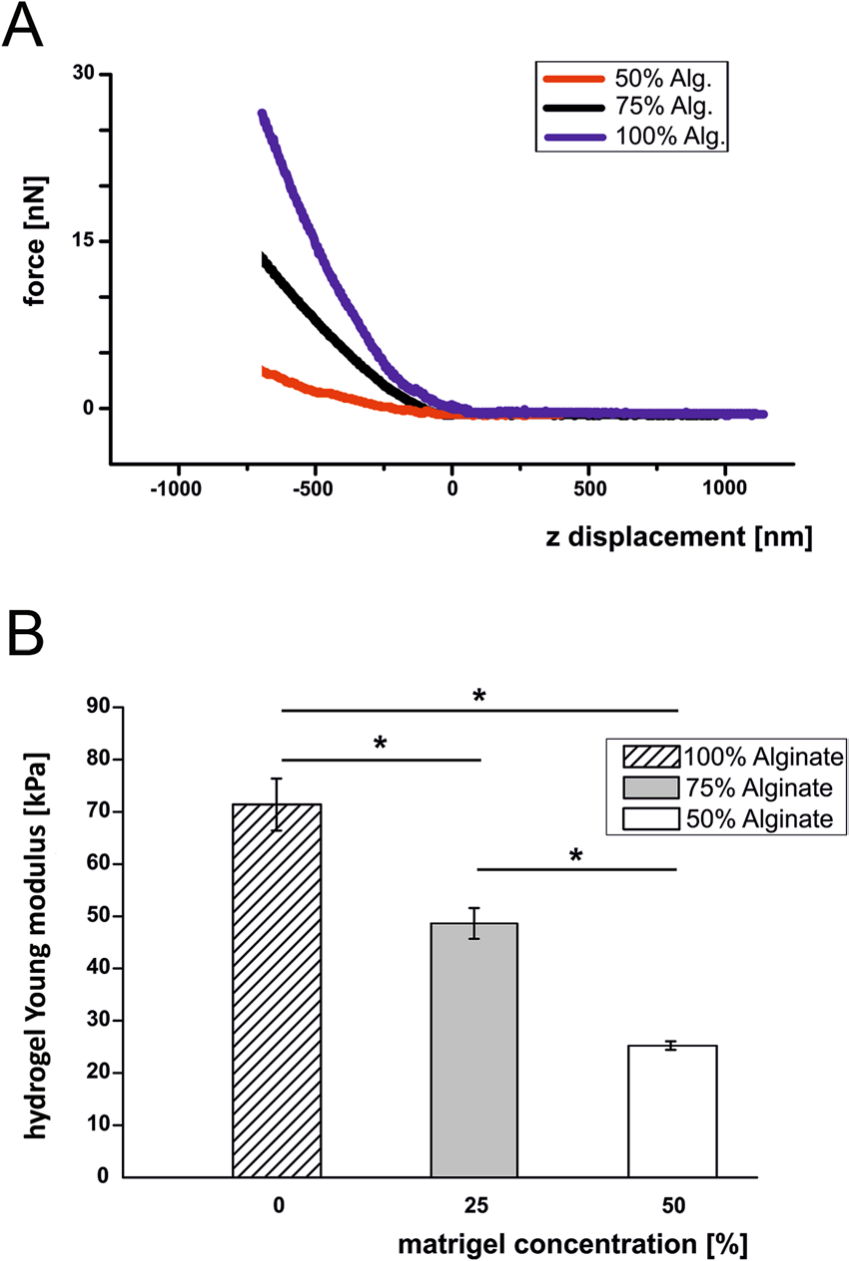
Hydrogel mechanical characterization. Panel A shows three representative force versus vertical displacement curves measured on three different hydrogels (100% Alginate, 75%:25% Alginate:Matrigel, 50%:50% Alginate:Matrigel). The z = 0 corresponds to the vertical piezo displacement where the AFM tip gets into contact with the hydrogel surface. Panel B shows the Young modulus average and standard deviation, STD) for the different hydrogels probed by AFM nanoindentation: bar colours correspond to different alginate concentrations, while matrigel concentration is shown along the x axis (Kruskal-Wallis test, p < 0.05).

The slope of the force curve after contact showed a negligible hysteresis between loading and unloading, which indicates a mainly elastic deformation of the hydrogels. Stiffness values were obtained using the same cantilever and the same approach-retract speed (4 μm/sec). The average force-distance curves for each hydrogel are displayed in Fig. 2, panel A. The curves show a qualitative, yet evident, difference in the compliance of the different hydrogels while deformed by the AFM tip.

Panel B in Fig. 2 reports mean values and standard deviation of the Young’s modulus measured over the hydrogel surface by AFM nanoindentation, following the procedure described in the methods section. The Young modulus varies significantly when changing alginate-to-matrigel concentrations (100% A, 75%:25% A:M, 50%:50% A:M), and, in particular, it decrease with the reduction of the alginate concentration. The Young modulus value of the sample with the higher concentration of alginate was in the range 66–76 KPa, while the sample with the lower concentration of alginate resulted softer (24–26 KPa). Our results demonstrate that hydrogel stiffness is dependent on alginate concentration. As reported by Samani *et al*.^47^, the Young’s modulus of an intermediate-grade IDC (Invasive Ductal Cancer) calculated on 21 samples is about 19.99 ± 4.2 kPa. This in totally in agreement with the stiffness of 50%:50% A:M gels.

### Viability and cell proliferation within gels

**Figure 3:**
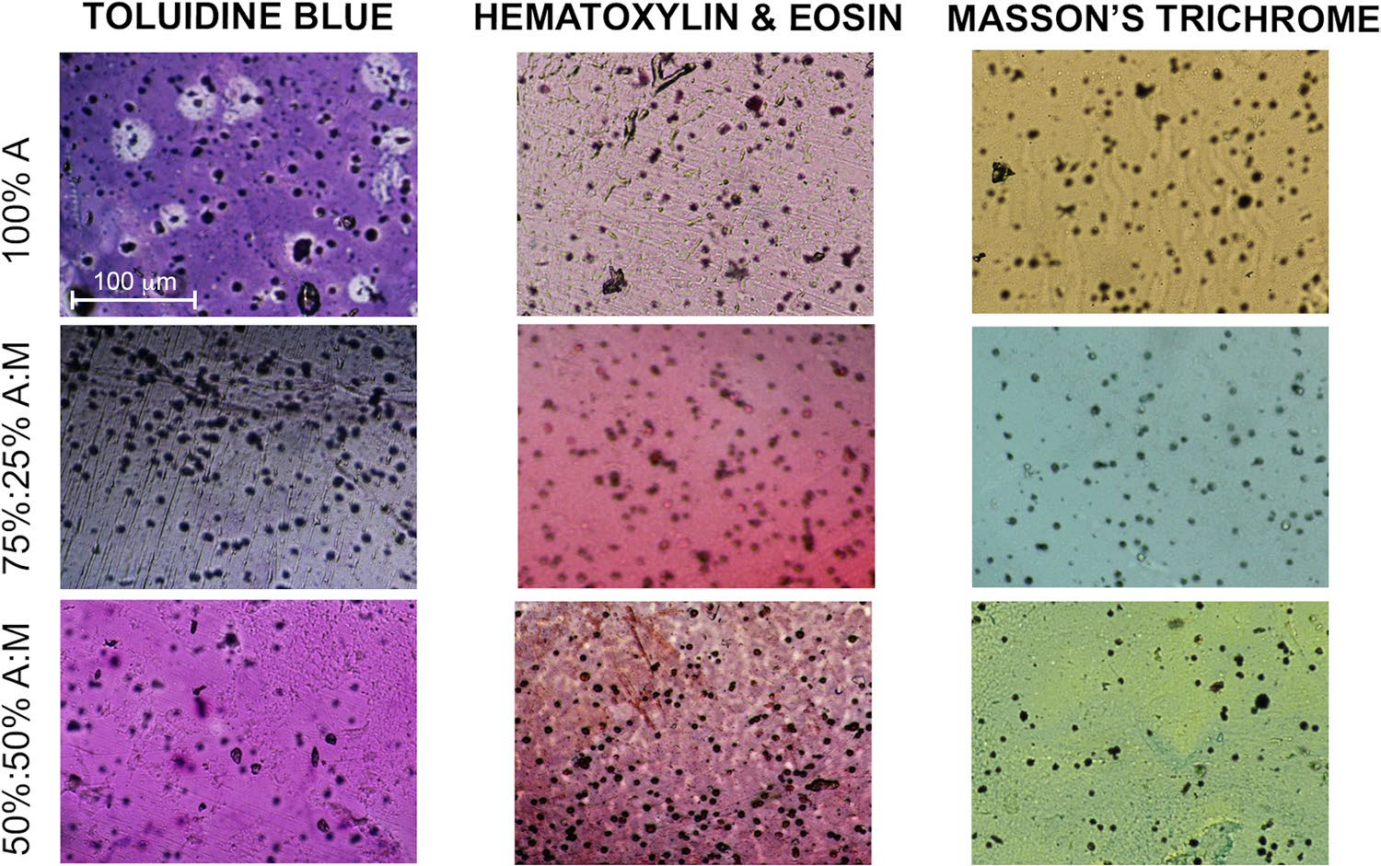
Histological analysis. - Immunohistological staining (toluidine blue, hematoxylin & eosine, Masson’s trichrome) and imaging of MDA-MB-231 cells (black) embedded within the three different composite materials. Images show no phase separation and homogeneous mixing in composite gels.

Immunohistological staining and imaging of MDA-MB-231 cells embedded within the three different composite materials highlight the good intimate mixing of Matrigel and Alginate (Fig. 3).

**Figure 4:**
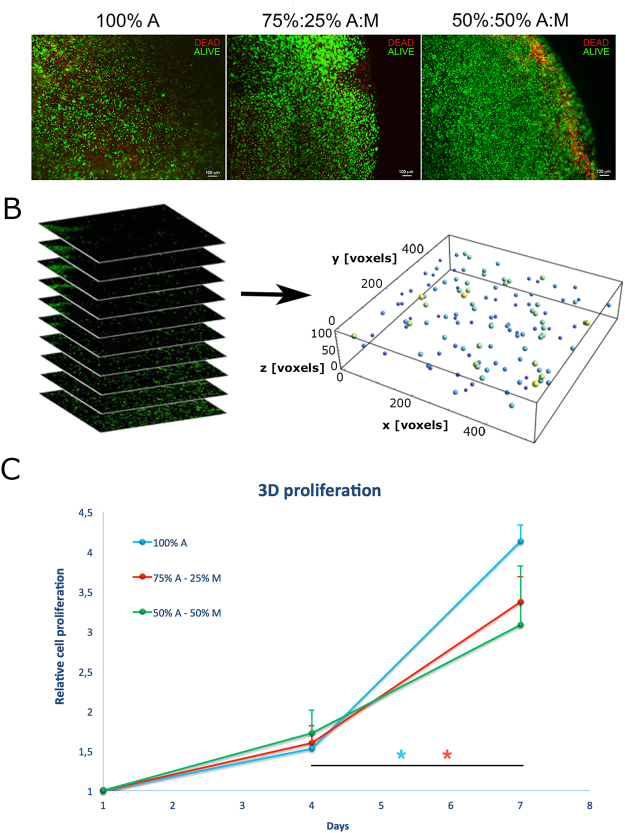
Cell viability and proliferation. (**A**) MDA-MB-231 cells cultured with materials for 24 h, stained with fuorescence dyes: calceinAM (green) for live cells and propidium iodide (red) for dead cells. (**B**) Procedure for nuclei segmentation to count cell number within different materials at different time points (**C**) MDAMB- 231 cell proliferation obtained by nuclei segmentation after 4 and 7 days of culture for different materials. Symbol *indicates statistical significance (ANOVA test; number of samples: N ≥ 10; p < 0.05).

Tumour cells in the hydrogels demonstrated high viability and density (Fig. 4, panel A) also in the inner parts of materials and time-dependent growth consistent with observations from confocal images (Fig. 4, panel C), validating our gelation protocol and gel mass transport properties. Although initial cellular proliferation in gels on day 4 was marginally low, it recovered to above 4 fold change relative to the initial cell number on day 7 (number of samples: N ≥ 10).

### Cell morphology: nuclear fragmentation, invadopodia, cytoskeleton irregularity and elongation

**Figure 5:**
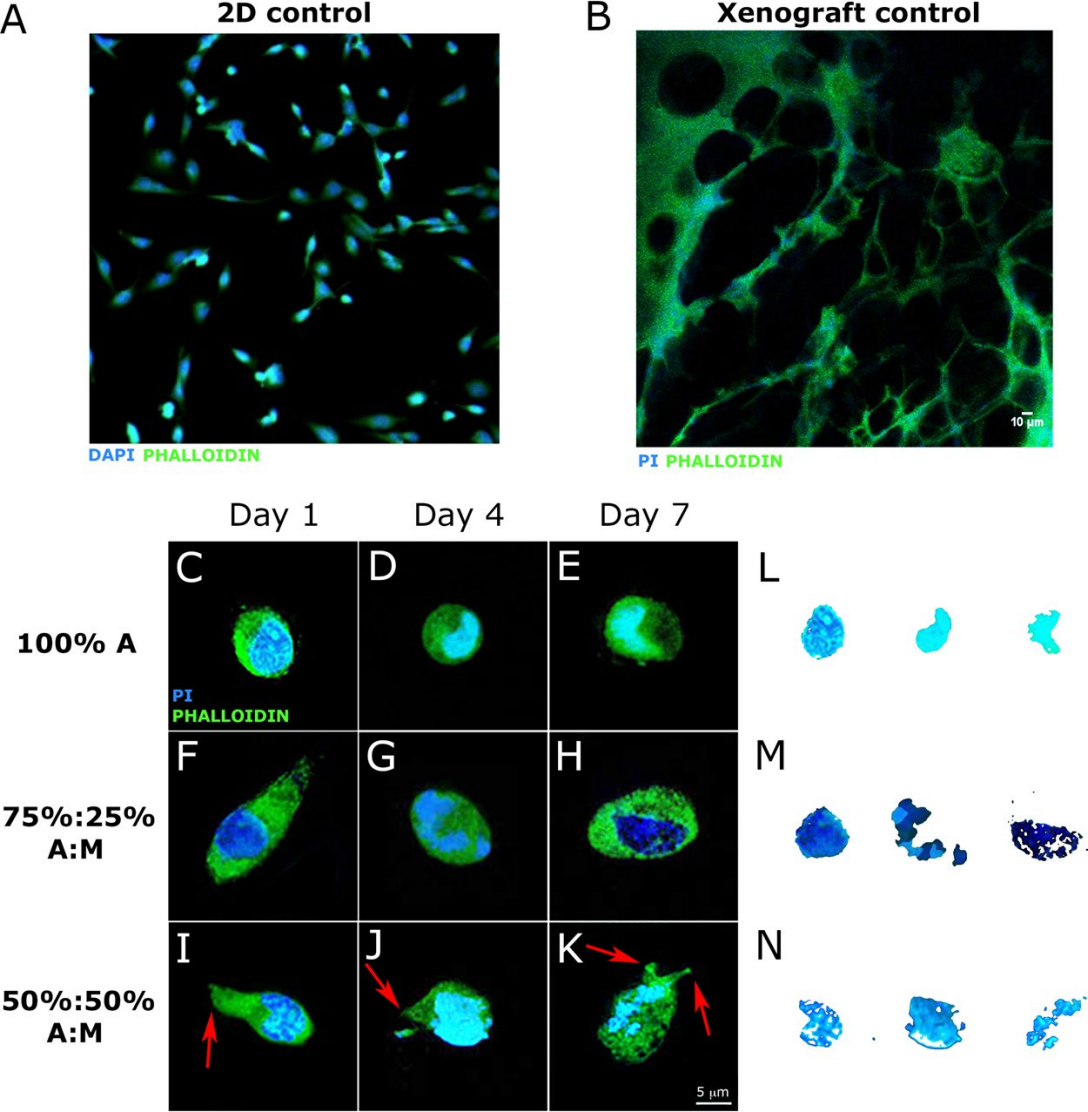
Cell morphological characterization. (**A**) Morphology of MDA-MB-231 breast cells cultured in two-dimensions. Cells were stained for F-actin (phalloidin) and nuclei were counterstained with DAPI; cells show a stellate morphology. (**B**) Confocal section of representative xenograft implant of MDA-MB-231. Cells were stained for F-actin (phalloidin) and nuclei were counterstained with Propidium Iodide. Cells show elongated/stellate morphology. c)-k) Confocal sections of representative cells embedded within Alginate/Matrigel composite gels at different time points (1, 4 and 7 days). Cells were stained for F-actin (phalloidin) and nuclei were counterstained with Propidium Iodide. Cells show round morphology in 100% A gels, thus not expressing their spreading capability; they show elongated morphology in 75%:25% A:M gels; they finally show stellate morphology and invadopodia (red arrows) in 50%:50% A:M gels. l-n) Nuclei shapes isolated from c-to-k panelsvshow that in presence of Matrigel cells express nuclear fragmentation, characteristic of malignancy.

Cell morphology was firstly qualitatively assessed. Both in 2D and *in vivo* (xenograft) conditions, MDA-MB-231 cells expressed, as expected, their typical elongated and stellate morphology (Fig. 5, panels A and B).

To obtain a similar conformation also in a 3D environment, we analysed cell morphology within the three different categories of gels at three different time points (1, 4 and 7 days; number of samples: N ≥ 7 per gel type).

In alginate gels, cells maintained a round morphology, typical of suspension phase and expressive of a non-malignancy condition, regardless of time point (Fig. 5, panels C, D, E); in presence of Matrigel, cells tended to elongate, accordingly to their aggressiveness (Fig. 5, panels F to K). More interestingly and in contrast to 2D controls, in the same gels we observed a nuclear fragmentation, known to be linked to malignancy as well. Indeed, while nuclei assumed a round and regular shape in 100% A gels (Fig. 5, panel L), they showed a multiple and jagged conformation in presence of Matrigel (Fig. 5, panels M and N). Finally, only in 50%:50% A:M gels, we observed manifestation of invadopodia (Fig. 5, panels I to K), actin-based protrusion of the plasma membrane through which cancer cells anchor to the Extracellular Matrix and degrade it. This feature, rarely observed in a 3D *in vitro* environment, was expressed by MDA-MB-231 in the same gels regardless of time point.

**Figure 6:**
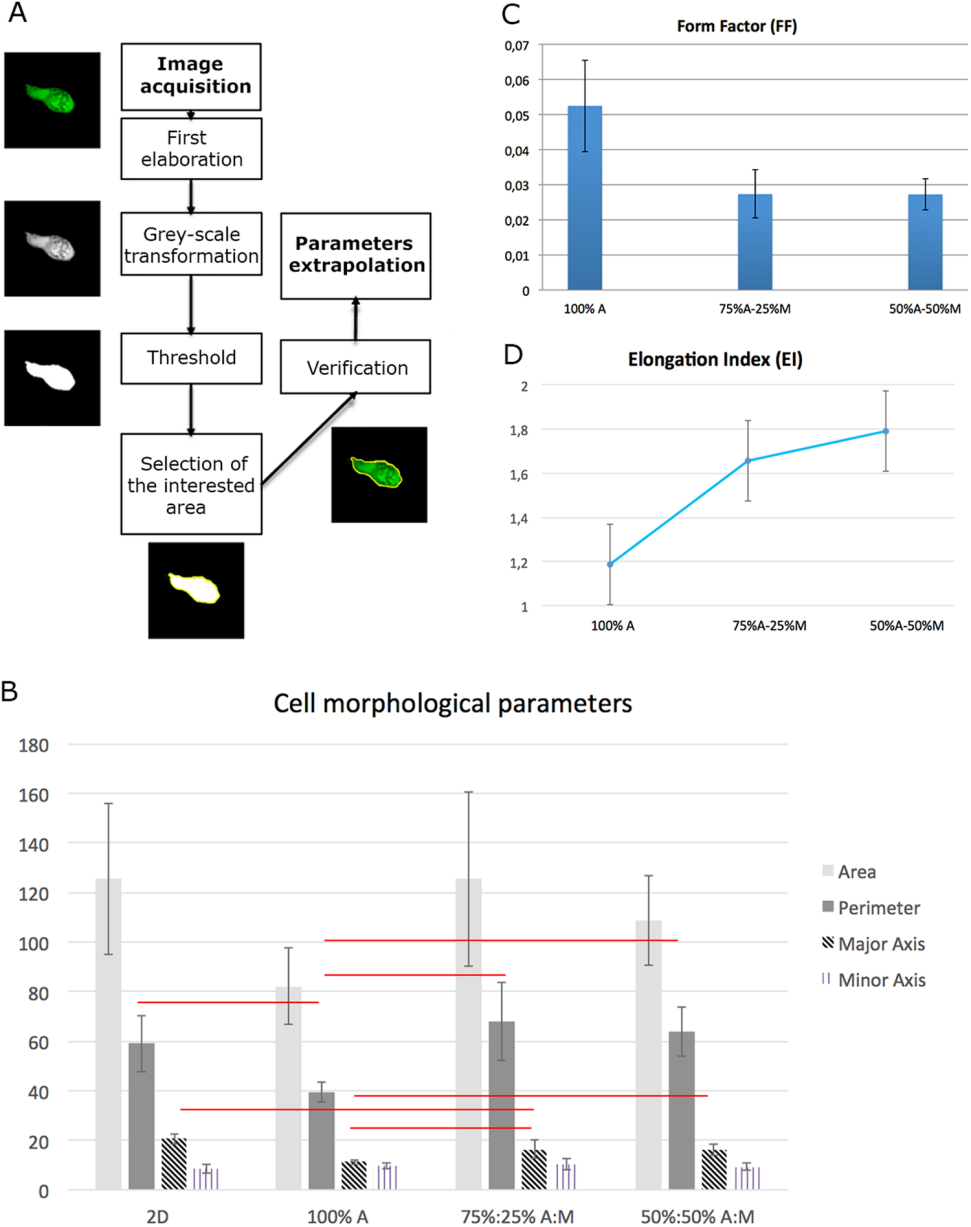
Morphological parameters extrapolation. (**A**) The extrapolation of cell morphological parameters follows different main steps. In the first step (top), images are adjusted through a series of transformation, i.e. brightness, contrast, grey-scale transformation and threshold. The area of the cell is then automatically selected, but a manual check is done by overlapping cytoskeleton (actin) image. Finally, parameters such as area, perimeter, major and minor axes are automatically extrapolated by the software. (**B**) Area, Perimeter, Major Axis and Minor Axis (averages, standard deviation) of cells cultivated on 2D substrates and within the composite gels. Red lines mean statistical significance (ANOVA test; number of samples: N ≥ 10; p < 0.05). (**C**) Form Factor (FF) for cells within the gels. Higher the IF, greater the irregularity of cell shape (**D**) Elongation Index (EI) for cells within the gels. Higher the EI, greater the elongation of cell shape.

From a quantitative point of view, gels containing a Matrigel component showed morphological features closer to elongated cells cultured in 2D conditions (Fig. 6, panel B). Irregularity (Fig. 6, panel C) and elongation (Fig. 6, panel D) resulted proportional to Matrigel content, highlighting that 50%:50% A:M gels are the most permissive to cell arrangement and organization in a 3D environment. In detail, irregularity is inversely proportional to Form Factor, since a value of Form Factor closer to $\frac{1}{4\pi }$ means that cells exhibit a most regular shape.

### Cell motility in bioreactor

**Figure 7:**
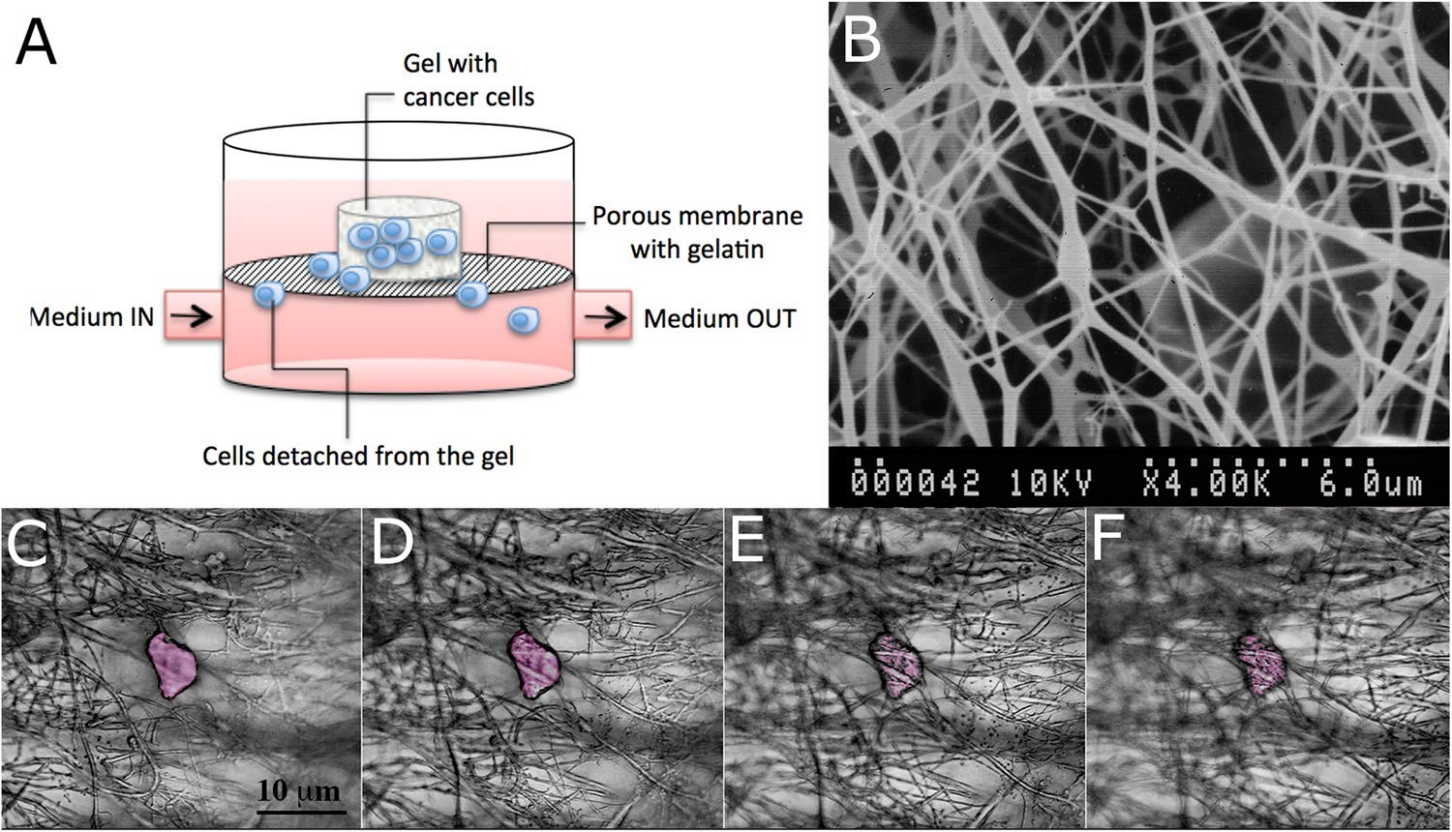
Preliminary steps of metastasis in bioreactor. (**A**) Schematic representation of the set-up for observation of cell spread and intravasation. Bioreactor was developed by React4life S.r.l. (**B**) Electrospun membrane used to mimick blood vessel interface. The membranes were realized in polycaprolacton (PCL) by electrospinning technique. (**C–F**) Confocal slides of cell entrapped within the membrane (cell is in false colours).

After 3 days in culture, membranes were removed from bioreactor and analysed under microscope to observe the presence of entrapped cells. At the same time, the media underlying the membrane (in the lower part of bioreactor) was observed to have a quantification of the number of cells passed after 3 days. Our results show that in all gels cells were able to migrate through the gels, escape from them and adhere to the porous electrospun PCL-gelatine membranes (Fig. 7, panels C to F), finally to migrate in the lower part. The number of cells passed after 3 days is reported in Table 1. The 50%:50% A:M gel resulted the more permissive to cell motility.

**Table 1:** Number of cells that passed the membrane in bioreactor after 72 hours of experiment were counted through a scanning of the plate and are reported in the table. The 50%:50% A:M gel resulted the more permissive to cell migration.

	100% A	75%:25% A:M	50%:50% A:M
*Number of cells passing the membrane after 72 hours*	**35**	**39**	**45**

## Discussion

It is an established fact that 3D cultures are essential for a better comprehension of cell behaviour, as they recapitulate an environment more similar to the *in vivo* one^48^. This is particularly important in pathological conditions, such as in cancer studies (e.g. breast cancer, the most common tumour in women). It has been long acknowledged that the microenvironment plays a role as a regulator of tumor progression^49^, and recent studies have illuminated that instead of 2D culture, the 3D matrix of animal-derived biomaterials such as Matrigel can significantly mediate the breast-cancer phenotype. Many researchers have used 3D matrices to study the migration of human breast cancer cells^50^; others, such as Bissell and colleagues, performed a variety of experiments using 3D culture systems for years and their work has shown that changing the ways cells interact with their 3D environment can significantly alter their phenotypes^51–53^.

However, at the moment no established 3D models with clinically relevant size and features exist to carry out standardized and reproducible studies on breast cancer and, most importantly, on its metastatic spread.

Authors previously demonstrated that alginate is a good candidate for the realization of 3D cultures of breast tumour cells^29^. In detail, we proved that alginate with controlled stiffness supported the proliferation of a specific lowly aggressive breast cancer cell line (i.e. MCF-7) and their arrangement in in the typical cluster-like organization. We adopted the alginate concentration (i.e. 0.5%) and the crosslinking density (i.e. 0.5 M) found in our previous work as starting points for the development of the composite gels here presented.

In the current work, we would like to take a step in the development of breast cancer models, proposing a new model of highly aggressive breast cancer; for this reason, we adopted a highly metastatic cell line, MDA-MB-231. However, to verify the migration potential of these cells in a 3D *in vitro* model, we combined alginate with a more permissive material allowing cell spread. We chose Matrigel because of the huge literature in support of this material and because it represents a standard for cancer cell culture.

Considering the societal relevance of metastasis and the poor scientific knowledge about the metastatic onset, there is a strong need of new 3D models of highly aggressive breast cancer, allowing cells expressing some features characteristic of their aggressiveness and manifesting that capacity of motility at the base of the metastatic cascade. Invasiveness assays usually adopt a thin layer of reconstituted Matrigel in Boyden chambers as very rapid, easy and inexpensive test that can be used to detect the migratory activity associated with matrix degradation and quantify the invasive potential of cells^54^. Because of the rapid degradation of Matrigel, this assay usually lasts few hours and Matrigel can’t be used as 3D substrate maintaining cells entrapped in.

We first designed five different hydrogel compositions (i.e. 100% A, 75%:25% A:M, 50%:50% A:M, 25%:75% A:M, 100% M), finally excluding 25%:75% A:M and 100% M because of a weak structural robustness and a low resistance in culture during the 7 days of culture.

The remaining three categories were characterized and validated from a mechanical and biological point of view, firstly assessing cell viability and proliferation both within the outer and inner parts of gels. Results show that cells were able to proliferate up to 4 times in all gel types; although cells within gels do not experience the same adhesion they have in 2D, it is known that tumour cells, and in particular MDA-MB-231 cells, are resistance to anoikis^55^. The sight difference subsisting in favour of alginate substrates may be attributed to the capability of cancer cells to easily migrate within the Matrigel and thus to more easily escape from Matrigel-based gels, as confirmed by the greater quantity of cells migrated in the lower part of the bioreactor. Confocal microscopy tools and 3D reconstruction techniques were used to analyse the morphological features of the cells, highlighting those differences related to their malignancy.

We observed that cells maintained a spherical shape in 100% A gels, while they assumed elongated shape in presence of Matrigel; this last feature is in agreement with what is known in literature, according to which MDA-MB-231 cells assume a particular stellate shape related to their malignancy. Cell elongation and cytoskeletal irregularity proportionally increased with the amount of Matrigel in the gels. In 50%:50% A:M gels, irregularity of the cytoskeleton is enhanced by the formation of invadopodia, actin-based protrusion of the plasma membrane through which cells anchor to the extracellular matrix and degrade it. Cell nuclei expressed irregular shape and fragmentation exclusively in cells with elongated shape and proportionally to the amount of Matrigel. This feature is known to be linked to cell malignancy as well, allowing us to say that 50%:50% A:M gels allow MDA-MB-231 cells to express those typical features of their aggressive *in vivo* behaviour. It is very interesting to notice that these morphological features were expressed by cells only in those gels having the same mechanical properties of *in vivo* tumors, i.e. ~20 kPa, highlighting the importance of mechanical and not only chemical properties of the 3D environment.

A bioreactor-based set-up for cell intravasation was adopted to monitor cell ability to migrate out from the gel and enter in circulation. Our results show that in all gels cells were able to migrate through the gels, escape from them and adhere to a porous electrospun membrane functionalized with gelatine to promote cell attachment; however, in 50%:50% A:M gels, cells showed a greater tendency to migration.

Even tough one of the limitations with the current study is the absence of gene expression data, these results, combined together, allows us to say that a new 3D model of aggressive breast cancer (i.e. 50%:50% A:M) was provided. It is based on an hydrogel-based, 3D, culture method which allows human carcinomas to grow *in vitro* and to maintain many typical *in vivo* properties, including 3D architecture, nuclear fragmentation and invadopodia manifestation, expression of morphological differentiation and migration capability. With respect to the current state-of-the-art, this model has a 3D, clinically relevant size; the model can be used for a prolonged cell culture time thanks to the mechanical robustness provided by the alginate component and, at the same time, it is permissive to cell migration and motility thanks to the Matrigel part, overpassing the poor integrity and stability of the previously proposed models, as reported at the beginning of this discussion.

From our point of view these facts make our 3D breast tumour model and the bioreactor-based intravasation set-up superior to the previously proposed models and migration assays, which do not allow to culture cells in a 3D environment of clinically relevant size and, at the same time, biologically active. Indeed, if a close mimicry of the *in vivo* situation is desired, it should be taken into account that tumours are 3D, auto-consistent structures and that metastatic cells actively escape from the primary tumour before entering in circulation. For this reason, standard microfluidic platforms and organ-on-chip technologies, usually using free circulating cancer cells, are poorly representative of the human context.

In conclusion, this 3D cell-laden hydrogel, combined with the presented bioreactor technology, is a good compromise between a reflection of the *in vivo* situation and manageable experimental effort, finally providing a completely new approach for studies on invasive breast cancer and drug testing.

## Methods

### Cell culture

Commercially available human MDA-MB-231 cells were used. MDA-MB-231 is an adherent cell line derived from pleural effusion of primary breast adenocarcinoma.

Cells were cultured in DMEM medium supplemented with 10% Fetal Bovine Serum (FBS) and 1% penicillin-streptomycin (P/S) (all from Sigma Aldrich), hereafter referred to as complete medium. Same cells were used both for 3D and 2D controls.

### Tumour hydrogel preparation and culture

To prepare alginate solution, alginate powder (Manugel GMB, FMC BioPolymer; viscosity: 200 m*Pa*s in a 1 wt%; particle size: 250 μm) was mixed in physiological buffer at a concentration of 0.5% (w/v), as previously assessed^29^. Effective intimate mixing was guaranteed by exposing alginate solution for 12 hours under vigorous magnetic stirring at room temperature. Matrigel was thawed over night at 4 °C. Meanwhile, an agar solution at 1% (w/v) concentration was prepared by mixing Agar (DIFCO Laboratories) in physiological buffer enriched with 0.5 M of CaCl_2_ (J. T. Baker). The solution was brought to the boil, poured into 6-well plates until ~1 cm height was obtained and allowed to cool until complete solidification. Holes of 0.5 cm diameter were then cut into the agar, using a Pasteur glass pipette, to make molds.

Cells were enzymatically detached from tissue plates and suspended in alginate solution. The suspension was moved to ice and Matrigel was added in variable quantity depending on the composite. Initially, we tested the following combinations: (i) 100% alginate (hereafter 100% A); (ii) 75% alginate and 25% Matrigel (hereafter 75%:25% A:M); 50% alginate and 50% Matrigel (hereafter 50%:50% A:M); 25% alginate and 75% Matrigel (hereafter 25%:75% A:M); 100% Matrigel (hereafter 100% M). The final cell density was 1 million/ml for all gel types.

To form a single gel, 100 μl of solution containing 100.000 cells were dispensed into agar molds using a tip. Gelation was allowed to take place at 37 °C for 1 hour and 15 minutes, in order to ensure the complete diffusion of calcium ions from agar to alginate solution and the thermally induced cross-linking of Matrigel.

Gels were gently removed from the molds, transferred into a 96 multi-well plate and maintained in complete medium containing 5 mM CaCl_2_. Medium was changed every two days. A schematic representation of the protocol is shown in Fig. 1, panel A.

### Atomic force microscopy

Stiffness measurements of hydrogels with a different alginate-to-matrigel concentration (100% A, 75%:25% A:M, 50%:50% A:M) were performed using a commercial AFM, equipped with a scanner that has a vertical range of 9 μm (Keysight Technologies, model 5500ILM). To compensate for piezo nonlinearity, creep and hysteresis, the scanner continued to operate in a closed loop during all the experimental session. A rectangular micro-cantilever (CSG 11 type, NT-MDT, Russia) with a conical tip was employed, and its spring constant was calculated by monitoring the cantilever oscillation in air due to thermal noise, following the procedure described by Hutter and Bechhoefer^56^.

Standard force curves were recorded to evaluate hydrogel stiffness. The section of the curve produced after the contact between the cantilever and the sample was then considered for further analysis. The applied load for cantilever deflections was calculated by first converting the output voltage from the AFM four-segment photodetector into nanometers of deflection, and then by multiplying the deflection by the cantilever spring constant. The conversion factor was calculated by taking several force curves on a hard glass substrate each time the laser spot on the cantilever had to be adjusted, and by considering the reciprocal of the average slope of the constant compliance region of the curves. When using sharp conical tips, the load versus indentation curve was evaluated to extract the Young modulus of the sample using the model proposed by Oliver and Pharr^57^. All measurements were performed at a constant approaching/retracting speed of 4 μm/s.

Hydrogels were glued onto a Petri dish using a minimum amount of fast cyanoacrylate glue, and, during measurement, samples were kept in a buffer containing 5 mM CaCl_2_. Force curves were recorded over a square grid (5 × 5 μm), in order to take into account intra-sample heterogeneity. For all the samples, three maps of 16 × 16 curves were collected onto three regions randomly selected over the samples surface.

A custom-built software was used for processing the single force curves in order to detect the vertical displacement corresponding to the AFM probe-gel surface contact. Data were expressed as mean values ± standard deviation. Statistical analysis was performed with Origin 8.0 (OriginLab Corporation, Northampton, MA) using Kruskal-Wallis test. Figures were edited with Corel Draw 2017 (Corel Corporation, Ottawa, Canada).

All AFM measurements reported in this paper were taken with the same cantilever and the same experimental conditions. For this reason, the observed relative changes in stiffness among the three samples are not significantly affected by uncertainties due to, for example, the tip geometry or the hydrogel Poisson’s ratio (regarding the Poisson’s ratio of all tested hydrogels, we assumed a constant value of ν = 0.5, corresponding to an incompressible, rubber-like material). On the contrary, the calculated absolute values could be affected by the abovementioned uncertainties.

### Cell viability assay

After 24 hours, cellular response to gel interaction was investigated to prove the biocompatibility of the new composites. For this purpose, gels were washed with phosphate-buffered saline (PBS) and incubated with a Live/Dead staining (Live/Dead Cell Double Staining Kit, Sigma Aldrich) at 37 °C for 15 minutes. Gels were then imaged using an upright microscope equipped with transmitted illumination and epifluorescence (Eclipse Ni-U, Nikon) to discriminate live cells (calcein AM stained-green) from dead cells (propidium iodide stained-red).

### Histology

Considering the different methods and kinetics of cross-linking of alginate and Matrigel, after 7 days cell-laden gels were processed for histological analysis in order to observe the level of intimate mixing of the two materials in generating new composites and the distribution of cells within. Briefly, samples were dehydrated in ethanol scale, paraffin embedded, cross-sectioned (7 μm thick) at different levels and stained with Masson trichrome, Toluidine blue and Hematoxylin & Eosin. Images were acquired by using a Nikon H550L optical microscope.

### Confocal microscopy

To examine the morphology of cells within the gels, 3D samples were analysed by optical confocal laser-scanning microscopy. 2D plastic cultures and paraffin-embedded xenograft slices were used as controls.

3D gels were fixed with 4% paraformaldehyde after 1, 4 and 7 days.

Nuclei were stained with 1 μg/ml propidium iodide (PI), while actin filaments were stained with 100 μM Alexa Fluor 488 Phalloidin (both by Sigma-Aldrich). Before PI staining, gels were treated with 100 μg/ml Rnase for 30 minutes.

Images were acquired through a confocal laser-scanning microscope (Leica TCS SP5 AOBS) with a sequential image acquisition to avoid spectral cross-talk. Alexa Fluor 488 was excited with the 488 nm line of the Ar laser and its fluorescence was collected in a spectral window of 500 to 580 nm. For PI, 543 nm excitation wavelength and 600–700 nm spectral window emission were used.

For 3D gels, stacks comprising 100 optical sections, each with a 375 × 375-μm field of view and 512 × 512-pixel image matrix, were obtained through a depth of 100 μm.

Xenograft paraffin-embedded sections derived from orthotopic xenografts of MDA-MB-231 cells in mice were provided by the National Cancer Research Institute of Genoa (Italy). Shortly, Swiss nu/nu immunocompromised mice were purchased from Charles River (Calco, Como) and maintained in 12-hour dark/light cycles with water and food ad libitum. Animals were housed and maintained in the Animal Care Facility of the IRCCS San Martino-IST, accordingly to national and European regulations (D.L. 4/3/14 No. 26; 86/609/EEC Directive). All animal experiments were approved by the internal Ethic Committee and by the Italian Ministry of Health. A group of 27 six-week-old female mice were anesthetized with a mixture of Ketamine-Xylazine given intraperitoneally, the mammary fat pad of the inguinal fourth gland was exposed and 500.000 cells were injected in 10 μl of PBS using a disposable syringe with a 29 G needle. Animals were monitored daily and euthanized when tumors reached the size of 1200 mm^3^ and before any sign of suffering became detectable. Tumors were removed and frozen for genomic analysis^58^.

Sections of 50 µm were cut rehydrated, and stained with PI and Alexa Fluor 488 Phalloidin.

### Image post-processing

In order to aid visualization of the arrangement of nuclei and actin filaments, the registered images were processed using the public domain NIH ImageJ program. Signals due to nuclei or actin filaments were separated from that due to background on the basis of signal intensity and grey-scale morphology. The same program was used to obtain 3D rebuilding of gels by z-stack methodology.

### Nuclei segmentation and quantification

Cell proliferation rate within 3D materials was obtained by counting the number of nuclei within the reconstructed confocal stacks. To do that, we adopted a protocol of nuclei segmentation developed by other authors^59^, that combines the Lines-of-Sight (LoS) concept with a local adaptive pre-processing to separate apparently touching cell nuclei into approximately convex parts representing single cell nuclei. Numbers of nuclei at days 4 and 7 were finally normalized on nuclei at day 1, in order to be presented as proliferation rates. The procedure of nuclei segmentation is schematically represented in Fig. 4, panel B. Statistical analysis was performed using one-way analysis of variance (ANOVA; number of samples: N ≥ 10; p < 0.05).

### Size and shape cell analysis

Confocal images were analysed by ImageJ to measure and quantify several features characterizing cells growing in 3D conditions. In particular, we considered cell area, perimeter, major axis and minor axis lengths.

We finally defined two parameters to evaluate the grade of irregularity and elongation of cell cytoskeleton:

1 $$Form\,Factor\,(FF)\,=\,\frac{Area}{Perimete{r}^{2}}$$

2 $$Elongation\,Index\,(EI)\,=\,\frac{Major\,Axis}{Minor\,Axis}$$

For a perfect circle (cell with round shape) the FF value tends to $\frac{1}{4\pi }$.

Statistical analysis was performed using one-way analysis of variance (ANOVA; number of samples N ≥ 7; p < 0.05).

### Electrospinning of polycaprolactone membranes and scanning electron microscopy (SEM)

Electrospun membranes were produced by dissolving polycaprolactone (PCL) in 1:1 absolute ethanol:chloroform (Bio-Optica) to create a 20% (w/v) final solution. The polymer solution was loaded into a syringe (12 ml), and a 21 Gauge needle was attached. The syringe was securely fitted to a syringe pump-driver (Harvard Apparatus PHD 2000). The needle tip was connected to a high voltage power source (Gamma High Voltage ES50P-10W) operating at 7 kV^60^ and positioned 12 cm from the collection plate (covered with an aluminium foil)^61^. The PCL solution was delivered at a constant flow rate of 2 ml/hour for 4 h. Finally, the membranes were air-dried for 24 h to allow residual solvent to evaporate.

The microstructure and porosity of PCL membranes were evaluated with a scanning electron microscope (SEM Hitachi 2500) after metallization with gold by using a Polaron SEM coating system.

### Assembly of tumour hydrogel and membrane within bioreactor for recapitulating the metastatic condition

The set-up for cell intravasation was assessed by using the multi-organ bioreactor by React4life S.r.l. (www.react4life.com). This is a double-compartmental device having the peculiarity of hosting 3D engineered tissues in the upper chamber, strictly in contact with a porous membrane, eventually functionalized to better mimic the blood vessel environment.

The previously described electrospun membranes were sterilized by irradiating with ultraviolet (UV) light at a distance of 10 cm over night; after that, they were sterilely placed between two polycarbonate rings to define the useful experimental area, pre-conditioned by pipetting 1% w/v gelatine solution in PBS above and incubated for 15 minutes. Finally, gelatine coating was removed and membranes were washed in PBS.

One membrane per experiment was placed within the multi-organ bioreactor. The tumour hydrogel, laden with MDA-MB-231 transfected with GFP to facilitate their visualization over time (GFP Stable Cell Line, Creative Biogene, Cat N. CSC-RR0102), was placed in the upper part of the bioreactor in contact with the membrane, to allow cells escaped from the gel to functionally attach to the membrane and to eventually enter in the bottom part of bioreactor, that is connected to a fluidic circuit enabling their collection. The cellular migration and intravasation was monitored taking pictures of the passed cells after 72 hours, and observing the cells entrapped in the membrane after 3 days.
